# Hydrogel Improved Growth and Productive Performance of Mango Trees under Semi-Arid Condition

**DOI:** 10.3390/gels8100602

**Published:** 2022-09-21

**Authors:** Khalid S. Alshallash, Mohamed Sharaf, Ashraf E. Hmdy, Sobhy M. Khalifa, Hosny F. Abdel-Aziz, Ahmed Sharaf, Mariam T. S. Ibrahim, Khadiga Alharbi, Amr Elkelish

**Affiliations:** 1 College of Science and Humanities—Huraymila, Imam Mohammed Bin Saud Islamic University (IM SIU), P.O. Box. 5701, Riyadh 11432, Saudi Arabia; 2 Department of Biochemistry, Faculty of Agriculture, Al-Azhar University, Cairo 11884, Egypt; 3 Department of Biochemistry and Molecular Biology, College of Marine Life Sciences, Ocean University of China, Qingdao 266003, China; 4 Department of Horticulture, Faculty of Agriculture, Al-Azhar University, Cairo 11884, Egypt; 5 Soils and Water Department, Faculty of Agriculture, Al-Azhar University, Cairo 11884, Egypt; 6 Department of Biochemistry, Faculty of Agriculture, Ain Shams University, Cairo 11566, Egypt; 7 Department of Biology, College of Science, Princess Nourah bint Abdulrahman University, P.O. Box 84428, Riyadh 11671, Saudi Arabia; 8 Botany Department, Faculty of Science, Suez Canal University Ismailia, Ismailia 41522, Egypt

**Keywords:** *Mangifera indica* L., polymer, fruit quality, vegetative growth, yield, vitamin C

## Abstract

Nowadays, the production of new mango cultivars is increased in many countries worldwide. The soil application of hydrogel represents a novel approach in the fruit trees industry. This investigation aims to study the effect of adding hydrogel (as soil conditioner) on the growth and yield of Shelly cv. mango trees. The experimental groups were assigned to a control group and three other treated groups, including 250, 500, or 750 g hydrogel∙tree^−1^. The results demonstrated that all applications of hydrogel composite had higher vegetative growth parameters, yield, and fruit quality characteristics of Shelly cv. mango trees compared to the control. The treatment of 750 g hydrogel∙tree^−1^ had higher values of vegetative growth parameters such as the leaf area, shoot length and tree canopy volume, compared to the control group and the other treatments. Similarly, higher values for yield and fruit quality were observed in the treatment of 750 g hydrogel∙tree^−1^. In conclusion, different amounts of hydrogel agent can improve the production and fruit quality of Shelly cv. mango trees in arid and semi-arid conditions in a dose-dependent manner.

## 1. Introduction

Mango is one of the most common fruit trees of the *Anacardiaceae* family [1], which is ranked third among fruit trees after citrus and grapes regarding cultivated acreage and fruit production in Egypt [2]. Shelly cv. mango fruit is produced from a cross between Tomy Atkins and Keitt. Such cultivar fruit possesses a rounded shape without a peak or sinus. The stalk end of the fruit is shallowly sunken, and the apex is rounded [3]. Mangoes are a cash-crop yield due to their excellent adaptability to new reclaimed soil conditions [4], and ability to produce an acceptable yield under irrigation water farming settings with restricted availability [5].

Global warming and climate change represent dangerous phenomena that lead to drought and other negative impacts [6]. Egypt is one of the most impacted regions by climate change [7]. Many studies expected longer inter-annual variability and warming higher than the global average [8,9]. Furthermore, water availability throughout the vegetative and reproductive stages will probably decline [10]. Water limitation is one of the most significant abiotic stress factors that affect mango tree development and productivity in dry and semi-arid regions [5]. There are many efforts to boost the output of mango fruits production due to the increased global demand [11]. Therefore, many tropical and sub-tropical crops, such as the mango, need novel conservative irrigation systems in the future [5].

Soil modification represents a novel technique needed to allow mango to grow under deserted conditions [12]. Hydrogels have recently been introduced as water-retaining polymers in many crops and fruit trees [13,14]. Such polymer represents a soil conditioner which holds water and other plant nutrients and releases them to the plants when the soil around the plant’s roots starts to dry-out [15]. Hydrogel increases soil water holding capacity and reduces irrigation frequency [16]. This property is related to a three-dimensional polymeric network which assists absorption and holding a significant amount of aqueous solution [16,17], Thus, this polymer volumetrically increases soil water content. When the soil dries up in the existing hydrogel, the water is progressively given back to the roots [18]. The hydrogel performance depends on their chemical properties such as molecular weight [19] and formation condition, along with the chemical composition of the soil’s solution or irrigation water [15]. Hydrogels also encourage the production of soil aggregates and enhance soil structure [14]. The various hydrogel levels’ capacity to absorb and store water allowed them to decrease the impact of drought stress [20], enhance the vegetative growth parameters, and suppress the activity of the catalase and peroxidase enzymes [16]. Soils treated with hydrogel had higher water content around olive trees. Moreover, Mid-day stomatal conductance, shoot growth, and oil yield are increased by using hydrogel in the root zone of olive plants [21]. The addition of hydrogel as soil application at concentrations from 250 to 750 g∙tree^−1^ increases the growth parameter, fruit physical properties, and fruit yield of Murcott mandarin trees [22]. However, limited studies are available regarding the soil application of mango trees with hydrogel. Hence, the herein study aims to study the effect of hydrogel as soil conditioner on the growth and yield of mango trees under arid and semi-arid conditions in two successive seasons, and to explore the influences of hydrogel as root-watering-crystals on physical and chemical properties of mango trees during the growing season. These results can lead to improving the irrigation and water management of mango trees under an arid and semi-arid farm field environment.

## 2. Results and Discussion

### 2.1. Effects of Hydrogel on Vegetative Growth Parameters

**Figure 1:**
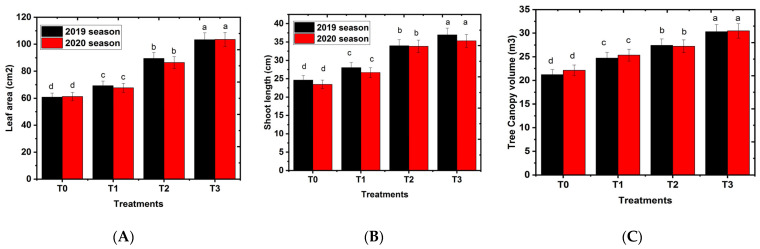
Effect of hydrogel soil application on vegetative growth parameters of Shelly cv. mango trees. T0, T1, or T2, or T3: 0, 250, 500, or 750 g hydrogel∙tree^−1^. (**A**) leaf area (cm^2^), (**B**) shoot length (cm), and (**C**) canopy volume (cm^3^). Bars indicate mean values ± SE (n = 9). Different letters above columns indicate significant differences among hydrogel treatments at *p* = 0.05, according to Bartlett’s test.

Effects of hydrogel on vegetative growth parameters are depicted in Figure 1A–C. The results demonstrated a clear response of Shelly cv. mango trees to the application of hydrogel on the farm. All hydrogel treatments had significantly higher values for all vegetative growth characteristics such as leaf area, shoot length, and tree canopy in the Shelly cv. mango in the studied seasons compared to the control group. By increasing the hydrogel level, the growth parameters were increased in a level-dependent manner. Hydrogel application was in coincidence with the cell expansion stage, which continued for a longer period compared to the control. The present result is confirmed by the previous work of Kaplan [23], who found that the application of hydrogel at a moderate concentration gave maximum growth parameters using a level of 500 g∙plant^−1^ apple ‘Gala Must’ variety. These results concur with those of Awad et al. [24] in the apple seedling and those of Cavalcante et al. [25] in passion fruit. In addition, by increasing the ratio of hydrogel in the substrate’s culture, Hüttermann et al. [26], Akhter et al. [27], and Chirino et al. [28] found increases in shoot height. In this study, as expected, the highest vegetative growth parameter values were found in the hydrogel at a concentration of 750 g hydrogel∙tree^−1^. However, these values decreased with low levels of the hydrogel. Our results are in the same line as those reported by Abobatta and Khalifa [15], who claimed that these super absorbent polymers (hydrogel) prevent water and nutrition materials from washing and therefore increase navel orange vegetative growth. The application of hydrogel increased all vegetative growth parameters. This result is related to the improvement of soil water and nutrient absorption by adding hydrogel. The nutrients taken by hydrogels from the soil are released by plants through an exchange relationship when they need nutrients for growth [1,23]. This action leads to improving the growth-boosting effect of hydrogel application. These outcomes align with those attained by Barakat et al. [29] in Banana Grad Nain cultivar using a 150 g∙plant^−1^ level of hydrogel. Similar trends were observed in Rabbiteye blueberry cultivar using a 20 g∙plant^−1^ level of hydrogel [30], and in banana cv. Grad Nain using a 1500 g∙hole^−1^ level of hydrogel [31]. The obtained results were agreement with those reporting that the vegetative growth of mango trees positively correlated with hydrogel quantity in the soil. Moreover, reducing hydrogel quantity for mango trees decreased the most mango growth parameters such as leaf area, shoot length, and tree canopy [5,32]. These findings may be due to the ability of mango trees to more efficiently photosynthesize under non-water drought conditions, which in return is reflected on the vigorous growth of the tree [33]. In addition, Wang et al. [34] stated that the water deficit of soil was sufficient to close the stomata in plant, which inhibited other physiological processes such as leaf enlargement and net photosynthesis. Moreover, trees that were grown in hydrogel produced plants with a high canopy volume, compared with soils free of hydrogel [35].

### 2.2. Effects of Hydrogel on Number of Fruits Per Tree

**Figure 2:**
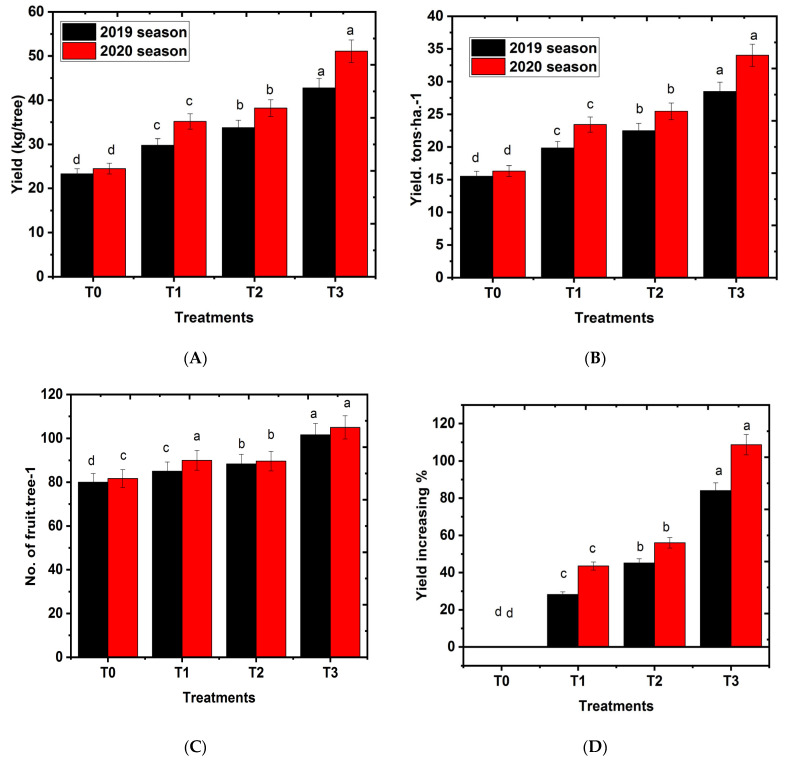
Effect of hydrogel soil application on fruit yield parameters of Shelly cv. mango trees. T0, T1, or T2, or T3: 0, 250, 500, or 750 g hydrogel∙tree^−1^. (**A**) yield·Kg·tree^−1^, (**B**) yield· Tons ha·^−1^, (**C**) No. of fruits∙tree^−1^, and (**D**) yield increasing %. Bars indicate mean values ± SE (n = 9). Different letters above columns indicate significant differences among hydrogel treatments at *p* = 0.05, according to Bartlett’s test.

The effects of hydrogel on the number of fruits per tree are depicted in Figure 2A–D. All hydrogel-treated groups had a higher number of fruits per tree in a level-dependent manner compared to the control group in both successive seasons. The higher value of fruit yield per tree is related to a high number of fruits retained per a tree at harvest compared to control. This result agrees with the study of Pattanaaik [36], who claimed that the yield of Assam lemon (*Citrus limon*) is increased by the application of hydrogel, compared to control trees. A similar trend was observed by Abdel-Aziz [22] in the Murcott mandarin. According to [26,37,38,39,40], soil increases the microbial activity and nutrient availability as a response to the beneficial effect of the hydrogel addition on boosting the quantity of fruits per tree. These results are consistent with Greven [41] and Moriana [27], who indicated a strong positive relationship between tree water status and olive output. In addition, the effect of irrigation on the number of fruits may be due to favorable conditions in the fruit set stage or a reduction in fruit drop [5]. Therefore, the hydrogel treatments enhanced the effective use of the irrigation water. Better resilience to the dry and semi-arid conditions on the farm is conferred by the higher irrigation water use efficiency [37]. In addition, mango cv. Keitt trees yield increased linearly with the increasing irrigation level because the higher fruit weight was produced in a high irrigation rate [38]. In this study, hydrogel increased the yield productivity of mango cv. Shelly, improved No. of fruits·tree-1, and promoted the parameters of the physical properties of fruit. Hydrogel can store water and conserve soil moisture, alleviate water stress, significantly improve the root-soil interface environment, and provide a good ecological environment for root growth [26]. At the same time, hydrogel increased soil moisture, organic matter, available nitrogen, phosphorus, and potassium contents, and enhanced root absorption and synthesis ability [39]. 

### 2.3. Effects of Hydrogel on Fruit Physical Characteristics 

**Figure 3:**
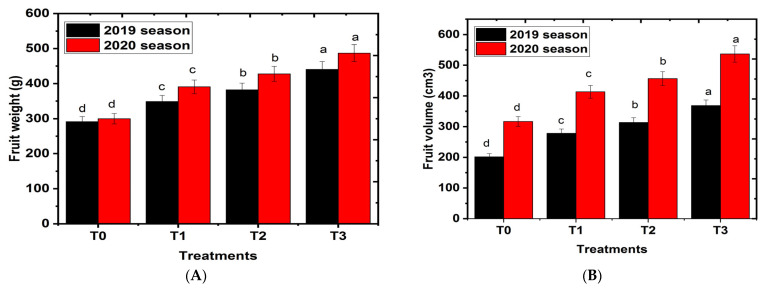

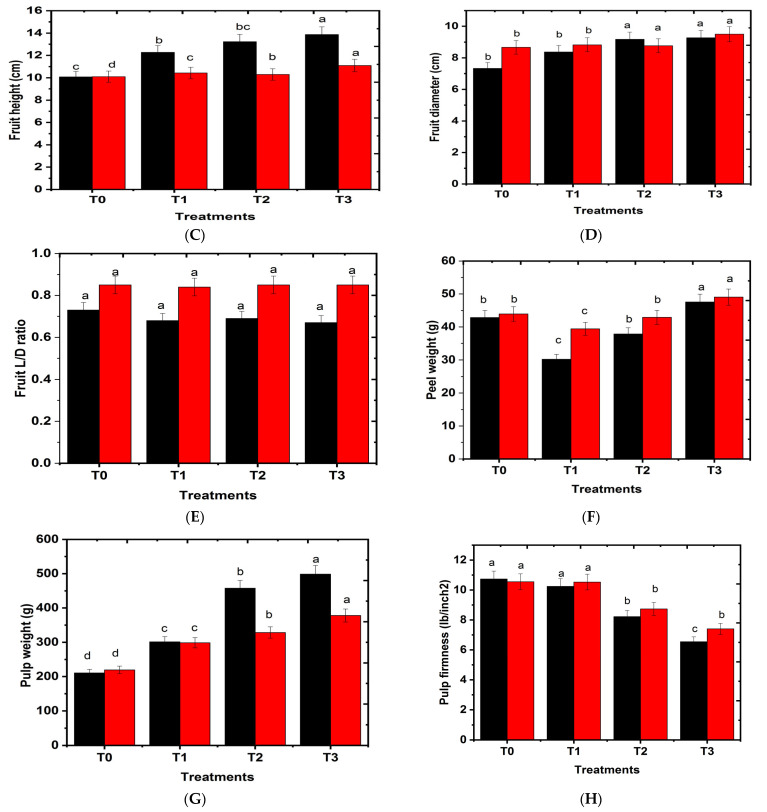
Effects of hydrogel soil application on fruit physical properties of Shelly cv. mango trees. T0, T1, or T2, or T3: 0, 250, 500, or 750 g hydrogel∙tree^−1^. (**A**) Fruit weight, (**B**) fruit volume, (**C**) fruit height, (**D**) fruit diameter, (**E**) fruit L/D ratio, (**F**) peel weight, (**G**) pulp weight and (**H**) pulp firmness. Bars indicate mean values ± SE (n = 9). Different letters above columns indicate significant differences among hydrogel treatments at *p* = 0.05, according to Bartlett’s test.

Effects of hydrogel on the fruit physical characteristics are shown in Figure 3A–H. All hydrogels had higher values of fruit weight, fruit volume, fruit diameter, fruit height, peel weight, pulp weight, and seed weight physical characteristics in a level-dependent manner compared to the control group. For fresh marketing, the increased fruit size is especially important [40]. In addition, the hydrogel treatments had lower fruit firmness in a level-dependent manner compared to the control group. According to Ahmad et al. [41] horticultural field practices during mango tree growth season have a significant impact on fruit physical properties such as fruit weight, volume, and shape. Madigu et al. [27] reported that the firmness of mango cv. Tommy Atkins maintained higher values with respect to deficit irrigation. There was a significant relationship between the water deficit and the reduction of cell size, and the reduction of water in plant tissues [28]. These results reflect that the highest average of some fruit physical parameters, such as fruit weight and fruit volume, recorded high values with an increase in hydrogel [2,42]. These results are in agreement with those reported by Nissi et al. [43], in sweet orange cultivar using a 2 kg∙acre^−1^ level of hydrogel. Similarly, Barakat et al. [44] found that adding a different level of hydrogel enhanced the fruit physical characteristics of Grand Nain banana cultivar compared to control, where the maximum values of physical characteristics were obtained when hydrogel was added to the soil at either 100 or 150 g∙plant^−1^, using different irrigation levels. In the present study, fruit physical characteristics demonstrated a significant overall hydrogel level. The cause may be that, in dry, hot climates with high evaporation, mulching and suitable supplementary irrigation can provide a good water, fertilizer, and aerothermal soil environment for crop growth. This environment is favorable for the synthesis of different amino acids, hormones, and other substances by the root system and promotes the root system’s capacity to absorb water and nutrients, enhancing photosynthesis and crop yield [45,46]. 

### 2.4. Effects of Hydrogel on Fruit Chemical Characteristics

**Figure 4:**
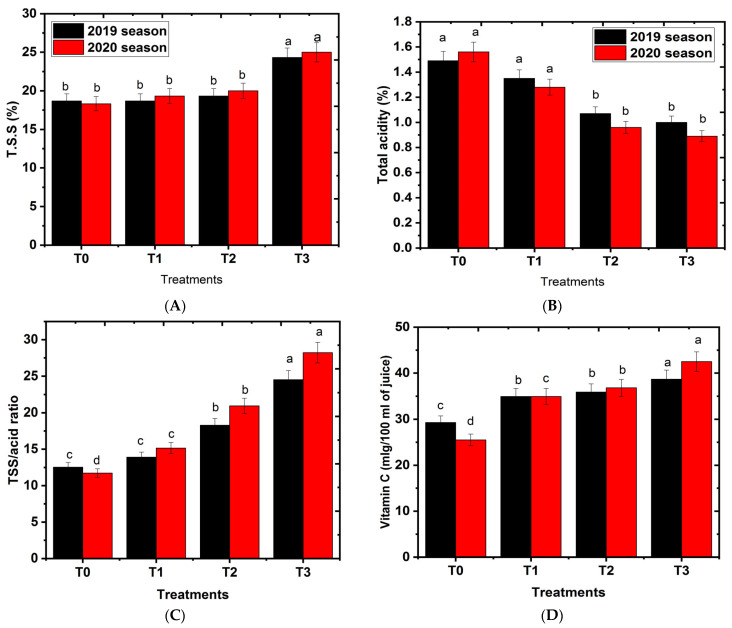

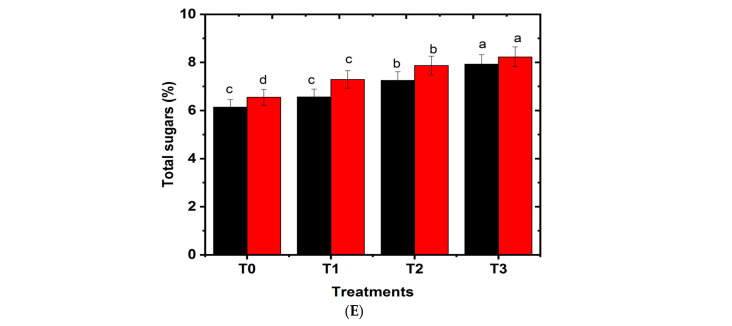
Effect of hydrogel soil application on fruit chemical properties of Shelly cv. mango trees. T0, T1, or T2, or T3: 0, 250, 500, or 750 g hydrogel∙tree^−1^. (**A**) TSS (%), (**B**) Total acidity (%), (**C**) TSS/acid ratio, (**D**) vitamin C, and (**E**) Total sugars (%). Bars indicate mean values ± SE (n = 9). Different letters above columns indicate significant differences among hydrogel treatments at *p* = 0.05 according to Bartlett’s test.

Effects of hydrogel on fruit chemical characteristics are indicated in Figure 4A–D. The higher values of TSS and total sugars were observed in 750·g∙tree^−1^ hydrogel compared to control and the other treatments, while the lowest values were recorded when trees were grown under the control treatment. Higher TSS values reveal better fruit quality [47]. These results are in harmony with those of Barakat [44], who found that the soil addition of hydrogel increases TSS of Grand Nain banana plants compared to a control group. Similarly, Costa [48] reported the lowest TSS values in Pacovan banana fruits. Moreover, El-Sayed [49] found that the soil addition hydrogel increases TSS% of Egazi olive trees compared to the control. In the current study, we noticed an insignificant difference among T0, T1 and T2 hydrogel treatments in TSS%. Therefore, adequate irrigation can reduce the soluble solids content of fruit through the dilution effect of soluble solids [50]. In the current study, trees in the control treatments had the highest values of acidity, followed in descending order by those treated with hydrogel in a level-dependent manner. In this regard, the least values of acidity were recorded by adding 750 g hydrogel∙tree^−1^ to the soil. Our results are in the same line as those reported by Abobatta [15], who found that the soil addition of hydrogel decreases total acidity %. A similar trend was observed in Washington navel orange fruits [51]. The increase in TSS and total sugars caused by hydrogel application may be due to the application of hydrogel, as the soil treatment enhanced the photosynthetic pigments in plants [52]. 

In the herein investigation, all hydrogel treatments had higher values in the fruit ripening index compared to control. The maximum T.S.S/acid ratio were obtained by adding hydrogel at 750 g∙tree^−1^ compared to the control and the other treatments.

**Figure 5:**
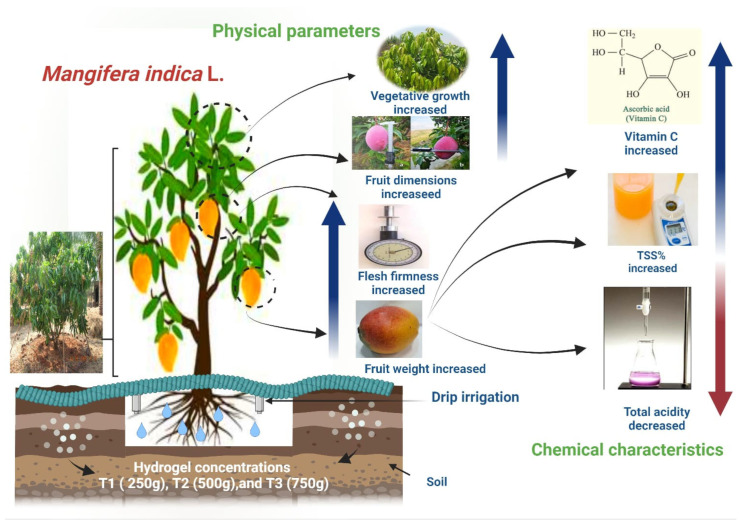
Summary figure to describe the beneficial action of hydrogel in Shelly Mango plants and fruits.

In the present study, most hydrogel treatments had higher levels of ascorbic acid content in fruit juice compared to control, where the highest ascorbic acid content in fruit juice was noticed in the treatment of 750∙g∙tree^−1^, as compared to the other treatments. These results are in line with Pattanaaik [36], who stated that adding hydrogel as a soil application enhances the ascorbic acid content in Khasi mandarin fruit [53]. From the above-mentioned results, it can be inferred that the application of hydrogel improved both the fruit’s physical and chemical properties, since the soil became wet for an extended period. Moreover, microbial activity and the availability of nutrients were increased [3], as shown in Figure 5.

**Figure 6:**
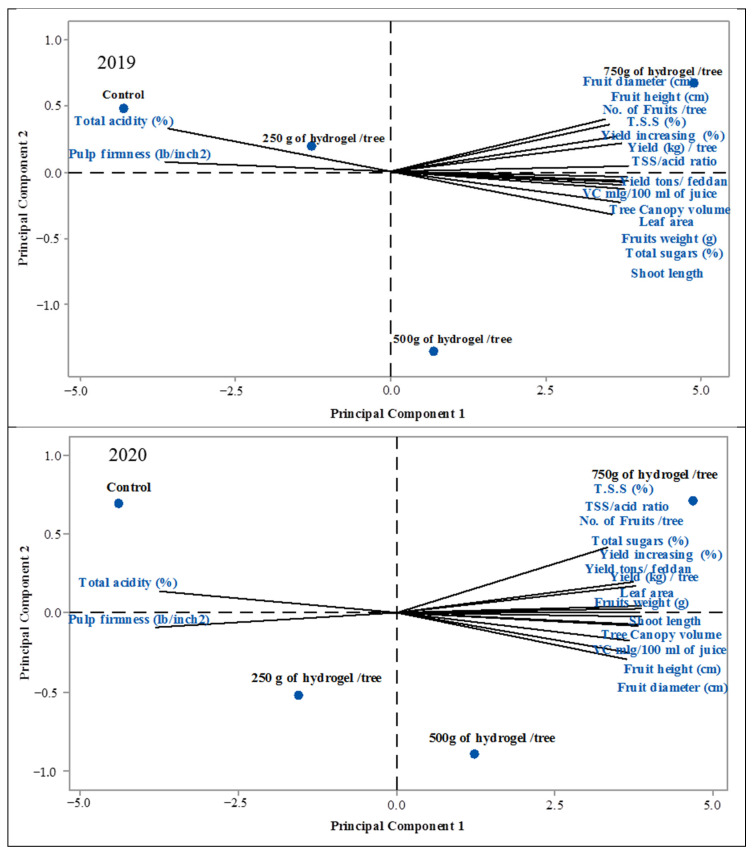
Biplot extracted by principal component analysis (PCA) among physical and phytochemical parameters studied based on different amounts of hydrogel treatments in 2019 and 2020 seasons: control, 250 g of hydrogel/tree, 500 g of hydrogel/tree, and 750 g of hydrogel/tree: Leaf area, Shoot length, Tree Canopy volume, No. of Fruits /tree, Yield (kg) /tree, Yield increasing (%),Yield tons/feddan, Fruits weight (g), Fruit height (cm), Fruit diameter (cm), Pulp firmness (lb/inch2), T.S.S (%),Total acidity (%), TSS/acid ratio, VC mL/100 mL of juice, and Total sugars (%) in 2019 and 2020 seasons.

**Figure 7:**
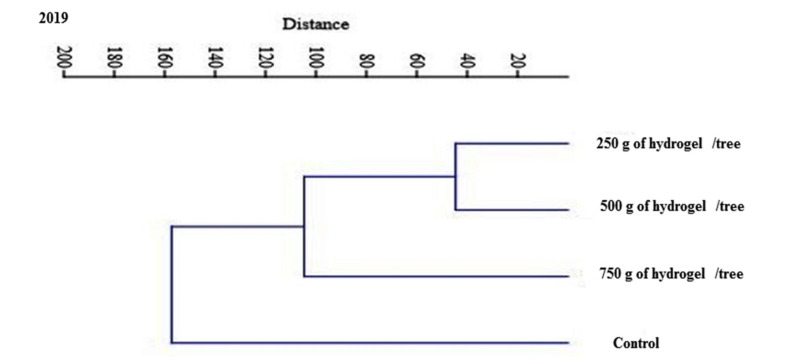

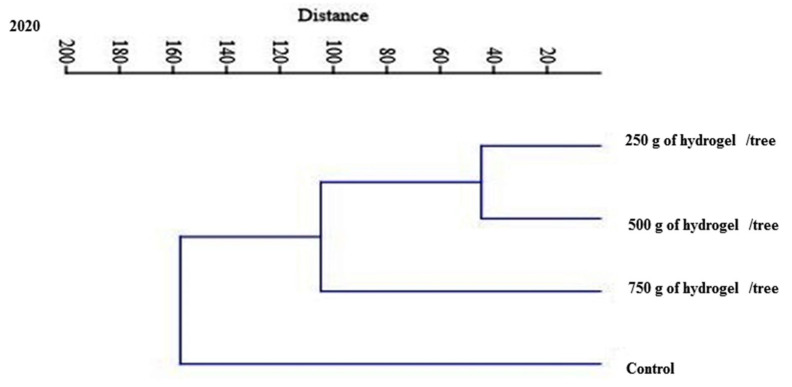
Dendrogram of cluster analysis for the effect of hydrogel soil application on physical and phytochemical parameters on growth and yield of Shelly cv. mango trees. Control, 250 g of hydrogel∙tree^−1^, 500 g of hydrogel∙tree^−1^ and 750 g of hydrogel∙tree^−1^: Leaf area, Shoot length, Tree Canopy volume, No. of Fruits /tree, Yield (kg)/tree, Yield increasing (%), Yield tons/feddan, Fruits weight (g), Fruit height (cm), Fruit diameter (cm), Pulp firmness (lb/inch^2^), T.S.S (%), Total acidity (%), TSS/acid ratio, VC mL/100 mL of juice, and Total sugars (%) in 2019 and 2020 seasons.

To evaluate the multivariate correlations between analyzed parameters, PCA is shown in Figure 6 and Table 1, which provide a summary of the individual values, variance %, cumulative percentage, and component loading. In successive periods of the study, only the first two primary components (PC1-PC2) had respective values larger than one (14.02 and 1.58) and (14.82 and 0.850). In light of this, data can be split into two factors that account for 97.64 percent and 87.00 percent of the total variation. For the first two elements in the seasons of the study, the figures were (87.78 and 9.94 percent), (92.75 and 5.30), and (87.78 and 9.94 percent), respectively (Figure 7).

## 3. Conclusions

The results of this study demonstrated that using soil conditioners such as hydrogels improved the soil’s ability to retain water. Adding hydrogel as a substance holding irrigation water at a concentration of 750 g∙tree^−1^ to the soil caused improved growth performance and productive quality of Shelly cv. mango. Therefore, hydrogel application is probably a promising agent to improve Shelly cv. mango production under arid- or simi-arid conditions. However, further research is needed to propose a particular hydrogel for different kinds of soil due to the variety in hydrogel performance.

## 4. Materials and Methods

### 4.1. Plant Materials and Experimental Design

This research was performed during two successive seasons in 2019 and 2020. The experimental groups were assigned to a control group and three other treated groups, including 250, 500, or 750 g hydrogel∙tree^−1^. The Shelly cv. mango (*Mangifera indica* L.) trees were grown in a private orchard located at Wadi Elmollak, Abu-Hammad city, El-Sharkia Governorate, Egypt (30°26′16.8″ N, 31°46′37.92″ E altitude). 

**Figure 8:**
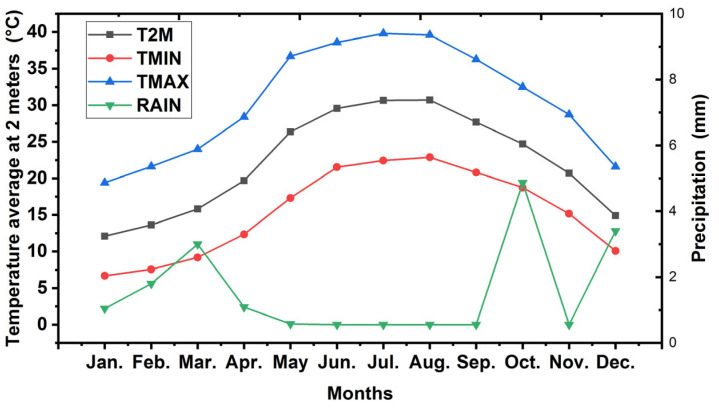
The average monthly temperature and precipitation during the growing season. T2M: Temperature Average at 2 Meters (°C) TMIN: Temperature at 2 Meters Minimum (°C) TMAX: Temperature at 2 Meters Maximum (°C) RAIN: Precipitation (mm).

After grafting onto Saber rootstock, the cultivar was planted in 2013. The trees were cultivated at 3 × 5 m apart (666 tree∙ha^−1^), and surface methods of drip irrigation were used in the research farm with eight adjustable discharge emitters/trees (8 L/h), through two irrigation lines. The region has a Mediterranean climate with an annual average temperature of 21.3 °C and an annual rainfall of 26 mm (Figure 8). The soil of the studied area is sandy (94.72% sand), as depicted in Table 2.

The soil conditioner hydrogel was applied to the soil at a depth of 20 cm on both sides of the trees in the middle of January throughout each season. We obtained hydrogel in the form of granules from the Directory General of Protected Cultivation, Agricultural Research Center, Giza, Egypt. These granules were prepared according to Farag [54]. The used hydrogel contained 1:5 (*v*/*v*) mixture of clay deposits (Bentonite), and polyacrylic super absorption polymers (SAPs).

The hydrogel had a pH of 7.12, bulk density of 0.67 g∙cm^−3^, real density of 1.72 g∙cm^−3^, total porosity of 61.0%, and water holding capacity of 60 cm^3^∙g^−1,^ according to Abobatta [15].

We formulated a mixture of chelated minerals containing 300 mg Fe, 150 mg Mn, 100 mg Zn, 50 mg Cu, and 50 mg B as the boric acid of the applied fertilizer. Such a mixture represented micronutrients in March, May, and August to the Shelly cv. mango trees and the recommended fertilization program (1000 g N, 1500 g P_2_O_5_, and 500 g K_2_O g∙tree∙year^−1^). According to the extension of the Egyptian Ministry of Agriculture, the suggested fertilization program was applied to all trees on an equal basis.

### 4.2. Experimental Measurements

#### 4.2.1. Vegetative Growth

Vegetative growth included average shoot length, leaf area, and tree canopy volume.

We estimated the average shoot length at the beginning of vegetative growth by tagging twenty shoots/tree. Four labeled branches (five shoots for every origin direction) were collected to measure the length of the shoot (cm) at the spring growth cessation in the studied cultivar.

The leaf area was evaluated in December of each successive season. The fifth distal leaf on the shoot, 20 mature leaves, were devoted and replicated three times. The leaf area (cm^2^) was then estimated according to the equation of Ahmed [55], as the following:

Leaf area (cm^2^) = 0.70 (L × W) − 1.06

where: L = maximum leaf length, and W = maximum leaf width.

Tree canopy volume was estimated according to the formula of Zekri [56] as follows:

Tree canopy volume (m^3^) = 0.52 × tree height × (diameter^2^)

#### 4.2.2. Tree Yield 

The harvest was completed within the normal commercial harvesting season on Sep. 21st at the maturity stage for each season (109 d from full bloom, according to Lavi [57]). The tree yield was recorded per kg. Increasing yield percentage was compared to the control according to the calculation of Abd El–Naby [58] as the following:

$$\mathrm{Yield}\;\mathrm{increasing}\;\left(\%\right)=\frac{\mathrm{Yield}\;\left(\mathrm{treatment}\right)-\;\mathrm{Yield}\;\left(\mathrm{control}\right)}{\mathrm{Yield}\;\left(\mathrm{control}\right)}\times 100$$

### 4.3. Fruit physical Parameters

At the harvest time, we selected samples of five fruits from each tree that were replicated three times in order to determine the parameters of fruit weight (g), fruit volume (cm^3^), fruit peel weight (g), fruit pulp weight (g), fruit height (mm), fruit diameter (mm), peel thickness (mm), and flesh firmness (lb./inch^2^) by using Digital force—Gouge Model IGV-O.SA to FGV-100A Shimpo instruments (Wilmington, NC, USA). These samples were then transferred in a bag to the subsequent chemical analysis. 

### 4.4. Fruit Chemical Characteristics 

The chemical characteristics of the fruits included fruit total soluble solids (TSS %), total fruit acidity %, fruit ripening index, ascorbic acid, and fruit total sugar content. The percentages of TSS and total fruit acidity were evaluated by combining 50 mL of distilled water with 10 g of fruit pulp from the Shelly cv. mango. A digital refractometer was used to estimate the TSS in filtrated juice. According to A.O.A.C. [59], the total acidity percentage was estimated using titration and expressed as citric acid. Fruit ripening index was calculated as follows: Fruit ripening index = Total soluble solids (%)/Total acidity (%). Ascorbic acid (vitamin C) was expressed as (ascorbic acid mg/100 mL juice) and was estimated by titrating juice samples with 2,6-dichlorophenol indophenol dye according to A.O.A.C. [59]. Fruit total sugar content was colorimetrically determined by dry fruit weight (g∙100 g^−1^ dry weight), according to the method of Chow [60]. 

### 4.5. Statistical Analysis

All data obtained during both seasons were analyzed using one-way ANOVA according to Ridgman [61] and Co-stat software according to Stern [62]. The principal component analysis (PCA) was performed to determine the multivariate relationships between the studied physical and phytochemical parameters using Minitab software Version 17.

## Figures and Tables

**Table 1:** Principal component analysis among—physical and phytochemical parameters studied based on different amounts of hydrogel treatments in 2019 and 2020 seasons.

	PC1	PC2	PC3	PC1	PC2	PC3
		2019		2020		
Eigenvalue	14.02	1.58	0.388	14.82	0.850	0.324
Variance	87.78	9.94	0.024	92.75	5.30	0.020
Cumulative	87.78	97.64	1.000	92.75	98.00	1.000
Components loadings						
Leaf area	0.264	0.106	−0.166	0.254	−0.09	−0.347
Shoot length	0.264	0.031	−0.226	0.242	−0.375	−0.205
Tree Canopy volume	0.267	−0.027	0.001	0.259	−0.074	0.090
No. of Fruits /tree	0.266	−0.007	0.132	0.251	0.261	0.130
Yield (kg)/tree	0.265	−0.081	0.103	0.258	0.050	0.159
Yield increasing (%)	0.258	−0.207	−0.055	0.258	0.050	0.158
Yield tons/feddan	0.255	−0.195	−0.279	0.258	0.051	0.160
Fruits weight (g)	−0.226	0.348	−0.481	0.254	0.050	0.266
Fruit height (cm)	0.096	0.741	−0.043	0.239	−0.148	0.122
Fruit diameter (cm)	0.261	−0.02	−0.342	0.233	0.421	−0.113
Pulp firmness (lb/inch2)	0.266	−0.007	0.132	−0.245	0.470	0.574
T.S.S. (%)	0.221	0.365	0.517	0.246	0.088	−0.241
Total acidity (%)	−0.260	−0.001	0.365	−0.242	0.318	−0.048
TSS/acid ratio	0.256	0.218	0.070	0.257	0.389	−0.24
VC mL/100 mL of juice	0.257	−0.196	0.195	0.251	−0.039	0.416
Total sugars (%)	0.265	0.099	−0.044	0.251	−0.114	0.132

**Table 2:** Physical and chemical properties of the experimental orchard soil.

Soil Depth (cm)	Soil Physical Analysis				EC (ds/m)	pH	Soil Chemical Analysis							
	Sand (%)	Silt (%)	Clay (%)	Soil Texture			Cations (meq/L)				Anions (meq/L)			
							Ca^++^	Mg^++^	Na^+^	K^+^	So4^=^	CI^−^	HCo3^−^	Co3^=^
0–30	94.72	3.00	2.28	Sand	0.50	7.0	2	1.11	1.5	0.21	0.82	2.0	2	00
30–60	93.72	5.00	1.28	Sand	0.47	7.2	2	0.9	1.17	0.22	0.29	2.0	2	00
60–90	92.15	4.65	3.2	Sand	0.40	7.4	2	0.8	1.04	0.20	0.34	1.6	2	00

## Data Availability

Not applicable.
